# Cigarette Smoking and Nephrolitiasis in Adult Individuals

**DOI:** 10.5812/numonthly.5251

**Published:** 2012-12-15

**Authors:** Mohammad Reza Tamadon, Mohammad Nassaji, Raheb Ghorbani

**Affiliations:** 1 Department of Internal Medicine, Fatemieh Hospital, Semnan University of Medical Sciences, Semnan, IR Iran; 2 Department of Infectious Diseases, Fatemieh Hospital, Semnan University of Medical Sciences, Semnan, IR Iran; 3 Department of Social Medicine, Semnan University of Medical Sciences, Semnan, IR Iran

**Keywords:** Adult, Nephrolithiasis, Risk factors, Smoking

## Abstract

**Background:**

Nephrolithiasis is a widespread multifactorial disorder. Constitutional, environmental and genetic factors play a role in stone formation. Although important advances have been made in understanding the pathophysiology of stone formation, none of the many theories have given a satisfactory explanation of this process.

**Objectives:**

The objective of study was to evaluate the probable relationship between cigarette smoking and nephrolitiasis in adult men.

**Patients and Methods:**

A total of 102 cases diagnosed with nepholithiasis and 121 age-matched healthy controls were recruited from June 2010 to September 2011. Smoking status and resultant data was obtained from both groups.

**Results:**

Twenty seven (26.5%) of the patients with stones and eighteen (14.9%) of the control group were current cigarette smokers. Our findings showed that smoking significantly increases the risk of nepholithiasis (OR = 2.06, 95% CI: 1.06-4.01, P = 0.034). There was no significant difference in the number of cigarettes smoked (P = 0.830) and years of smoking (P = 0.536) between subjects with and without stones (P = 0.536).

**Conclusions:**

This study suggests that cigarette smoking might be an independent risk factor for the development of nephrolithiasis.

## 1. Background

Renal stones or nephrolithiasis is a common challenge and affects populations worldwide. The worldwide prevalence of nephrolithiasis is between 2 and 20%. However, this prevalence varies by age, sex and race (1). Nephrolithiasis imposes a significant burden on human health and the economy. It is estimated that almost 50% of stone formers will have a recurrence within 10 years (2). A positive family history of nephrolithiasis is strongly associated with increased stone risk. Relative risk in those with a positive family history is 2.5 times higher (3).

Studies have shown that the prevalence of kidney stones is increasing in adults globally. The reasons for this increase are not clear. Changes in dietary habits, lifestyles and increased rates of obesity are thought to be contributing factors (4). In certain areas of the world, as in the Middle East, the lifetime risk appears to be higher (20% to 25%) (5). In Iran, the reported prevalence of nephrolithiasis is 5.7% and is more common in the central and southwest provinces (6).

Renal stone disease is a multifactorial disorder. Constitutional, environmental and genetic factors play a major role in the development of stones. Although important advances have been made in understanding the pathophysiology of stone formation, none of the many theories have provided a satisfactory explanation of this process (7).

Cigarette smoking is clearly identified as the chief preventable cause of mortality and morbidity in the world. Despite growing knowledge about the adverse health and economic consequences of smoking, the pervasiveness of smoking remains alarmingly high. Cigarette smoke contains more than 4,000 substances known to be antigenic, cytotoxic, mutagenic, and carcinogenic. Many of these substances produce significant physiologic effects on human body and can harm almost every organ (8).

It has been posited by some authors that trace elements such as magnesium, zinc, aluminum, iron and copper may have role in lithogenesis but this contention is still under debating. Probably some of them may contribute or, conversely, inhibit crystal nucleation of stone components (9, 10). The concentration of these trace elements and heavy metals in body tissues is elevated in cigarette smokers (11). Recent studies indicated that Lead and Cadmium induced glomerular dysfunction in smokers, possibly due to high serum levels in smokers than nonsmokers (12). Based on the above finding, smoking may affect stone formation. Some epidemiological data links smoking with the risk of stones (13, 14).

## 2. Objectives

Since nephrolithiasis is considered as a complex disease, identifying the pathophysiological mechanisms and the independent preventable risk factors could help decrease the number of patients suffering from this malady. The aim of this study is to evaluate a possible relationship between kidney stones and the smoking status of patients.

## 3. Patients and Methods

### 3.1. Patients

We conducted a case-control study from June 2010 to September 2011. Adult men (≥ 18 years old) who were newly diagnosed as having a stone in the upper urinary tract in nephrology clinic of Fatemieh Hospital during this period were enrolled as cases. This hospital is a university affiliated center for kidney diseases in Semnan, Iran. Nephrolithiasis was diagnosed by ultrasonography and radiography. Eligibility and final diagnosis was confirmed by a university nephrologist.

Age (± five years) matched controls were randomly selected from subjects receiving routine preventive health examinations at the same hospital during the same period. The controls had no history and no clinical and imaging finding of urinary stones. All study subjects lived in Semnan city In center of Iran.

### 3.2. Study Design

Written informed consent was obtained from each participant in this study. The project was approved by research committee of Semnan university of medical science. Patients with well-documented conditions that increased the risk of formation of stones including gout, primary hyperparathyroidism, renal tubular acidosis, diabetes mellitus, Crohn’s disease, chronic urinary tract infection, chronic diarrhea, renal failure and anatomical abnormality were excluded. Ultrasonography and radiogical studies were performed in order to exclude anatomical abnormality. All subjects were visited by a nephrologist and given a questionnaire which included questions about their age, the family history of stone disease and smoking status. The information about use of cigarettes was collected with regard to the duration of use, and the daily number of consumption. Subjects were defined as cigarette smokers if they had smoked five or more cigarettes per day for at least six months. Intermittent and former smokers were excluded.

### 3.3. Statistical Analysis

Data were analyzed by Kolmogrov-Smirnov, t test and logistic regression using SPSS 18.0.P-values of less than 0.05 were considered statistically significant.

## 4. Results

In this cross-sectional study, 102 male patients with kidney stones and 121 healthy control subjects were evaluated. The mean (± SD) age of patients was 42.5 ± 14.1 years and the controls were 42.8 ± 16.1 and showed no significant difference (P = 0.870). Age distribution in case and control subjects is shown in Table 1. First-degree relative family history of nephrolithiasis was positive in 42.2% (n = 43) of cases.

**Table 1:** Age Distribution in Case and Control Subjects

	Age, y					
	18-29	30-39	40-49	50-59	≥ 60	Total
**Nephrolitiasis +, No. (%)**	17 (16.7)	35(34.3)	19(18.6)	15(14.7)	16(15.7)	102 (100)
**Nephrolitiasis-, No. (%)**	25 (20.7)	35(28.9)	19(15.7)	22(18.2)	20(16.5)	121 (100)

Among the cases with stones, 27 (26.5%) of subjects were current regular cigarette smokers whereas in the control group 18 (14.9%) were smokers. The mean (± SD) number of smokers was 15.1 ± 9.5 in the cases and 14.6 ± 4.4 in the control group and was not significantly different (P = 0.830). The mean (± SD) duration of smoking was 19.1 ± 11.9 and 17.2 ± 95.5 years in patients and control subjects, respectively. There was also no significant difference in years of smoking between subjects with and without stones (P = 0.536).

Logistic regression analysis showed that smoking was significantly associated with urinary stones (OR = 2.06, 95% CI: 1.06-4.01, P = 0.034) (Table 2).

**Table 2:** Logistic Regression Analysis on the Effect of Smoking on the Risk of Nephrolitiasis

	β Coefficient	β SE	*P *value	OR	95% CI for OR
**Smoking**	0.72	0.34	0.034	2.06	1.06-4.01
**Constant**	-0.32	0.15	0.037	-	-

Abbreviations: CI,confidence interval; OR, odds ratio.

## 5. Discussion

The influence of smoking on human health is an important worldwide problematic issue. In recent years, special attention has been paid to different socioeconomic, alimentary, occupational, and environmental factors’ effects on the pathophysiology of urinary stone formation. One of the variables is smoking and a few published studies have examined the association between smoking and kidney stone disease (15).

In this study, the proportion of smokers among patients with nephrolithiasis was significantly higher than in the control subjects. Cigarette smoking was 2.06 times more common in stone formers than in the control group. Hence, smoking may be an independent risk factor for nephrolithiasis.

Urinary tract stones were found to be more common among workers chronically exposed to cadmium as compared to the general population (16). One of the possible factors which may explain the effect of smoking on stone formation is a high body cadmium and lead level in smokers. Cigarette smoking may induce urolithiasis by decreasing urinary flow and increasing serum cadmium in healthy subjects (17, 18). Also, Scott et al in their study proposed that increased serum cadmium levels associated with cigarette smoking may be a risk factor for urinary tract stone formation (19).

Another possible explanation is the increase in plasma arginine vasopressin level in smokers. Lower urine output is thought to be an important risk factor for urinary stone formation. Arginine vasopressin has a strong antidiuretic action (20). Mooser reported that signiﬁcant increases in plasma arginine vasopressin levels were associated with cigarette smoking (21). Therefore, cigarette smoking may further decrease urinary output which may be a potential trigger for stone formation.

On the other hand, smoking can increase production of reactive oxygen species and oxidative stress in the kidney, leading to renal injury (22). These injuries increase the nucleation, aggregation, and retention of crystals in the kidney thereby promoting the formation of stones (23, 24). Stones are more common in men than in women (2). Since smoking tends to be more common in men this may be one of the reasons for higher rate of stones in male sufferers.

In accordance with our finding, Liu et al. evaluated the impact of cigarette smoking and betel quid chewing on the risk of calcium urolithiasis. They showed that cigarette smoking (OR=1.66; 95% 95%CI: 1.11–2.50; P = 0.014) was an independent risk factor for the development of calcium urolithiasis (14). Similarly, Hamano et al. confirmed that calcium oxalate stones are strongly associated with several coronary heart disease risk factors which include smoking habits. In this study, they found cigarettes can increase the risk of urolithiasis by as much as 4.29-fold (25).

In contrast to our findings, Siojewski et al. in their study do not support the possible association between smoking, trace elements and urinary tract stones. They believe that further investigationis needed in this area (13). In another study, the associations between diet and the risk of kidney stones in male smokers was evaluated. Following5 years of follow-up, they concluded that smoking had not been found to be related to higher risk of lithogenesis (26).

Our findings showed that there was not a significant difference in number of cigarettes and the duration of smoking between the two groups. This may imply that smoking per se, versus duration and number, is a risk factor for stone formation. However, our study has several limitations. Firstly, our study sample was not large, thus limiting the variable of stronger statistical significance. Secondly, we did not do analysis to determine the type of stones. Smoking may affect the formation of a specific kind of urinary stone. Third, some confounding variables such as dietary habits were not matched between the two groups.

In conclusion, our findings implied that cigarette smoking may be a risk factor for urinary stone formation. Therefore, further studies, especially large population-based and with consideration of other etiologic factors, is needed to better clarify the possible effect of smoking on stone formation.
